# Origin of Bilinear Low Cycle Fatigue in Ti-6Al-4V Alloy: A Crystal Plasticity Study

**DOI:** 10.3390/ma18173931

**Published:** 2025-08-22

**Authors:** Haifeng Xu, Dianxi Yang, Wei Li, Zhengxiao Guo, Yinghonglin Liu

**Affiliations:** College of Mechanical Science and Engineering, Northeast Petroleum University, Daqing 163318, China; y1545902468@163.com (D.Y.); liweinepu@163.com (W.L.); guozhengxiao0727@163.com (Z.G.); honglin_7799@163.com (Y.L.)

**Keywords:** Ti-6Al-4V alloy, crystal plasticity finite element method, low-cycle fatigue, bilinear, life prediction

## Abstract

This study resolves the long-standing question of the origin of bilinear Low Cycle Fatigue (LCF) behavior in Ti-6Al-4V using a high-fidelity CPFEM-XFEM framework. We identify that the fundamental origin lies in a fundamental shift in the efficiency of converting macroscopic energy dissipation into microscopic damage. This energetic efficiency is directly governed by the evolution of plastic strain heterogeneity (quantified by the Coefficient of Variation, CV). At low strain amplitudes, high strain localization (high CV) creates a highly efficient “energy funnel,” concentrating dissipated energy into a few critical grains. This manifests physically as a single-crack failure mode, where the crack initiation phase is prolonged, consuming ~80% of the total fatigue life. Conversely, at high strain amplitudes, deformation homogenization (low CV) leads to inefficient, diffuse energy dissipation across many grains. The material must therefore activate a more drastic failure mechanism—multi-site crack initiation and coalescence—to accumulate sufficient damage, reducing the initiation phase to just ~45% of the total life. Therefore, the bilinear C-M curve is the macroscopic signature of this transition from an energetically efficient, localized damage mode to an inefficient, distributed one. This work provides a quantitative, mechanism-based framework for understanding and predicting the complex fatigue behavior of advanced metallic materials.

## 1. Introduction

With its exceptional combination of high strength-to-weight ratio, excellent fracture toughness, and corrosion resistance, Ti-6Al-4V alloy has become an indispensable critical structural material in the aerospace industry [1,2]. Particularly in core components such as engine fan disks and compressor blades, the alloy is subjected to typical Low-Cycle Fatigue (LCF) loading under complex start–stop cycles and varying operational conditions during its service life [3,4]. Consequently, LCF damage has emerged as the dominant factor governing the service life, safety, and reliability of these key components, making its accurate prediction a cornerstone of equipment integrity.

While the classic Coffin-Manson (C-M) relationship is widely used to characterize LCF behavior, extensive experimental studies have revealed that for many engineering materials, the C-M curve is not simply linear on a log–log scale. Instead, it often exhibits a distinct “bilinear” characteristic [5,6,7,8]. This phenomenon, first systematically documented by Radhakrishnan [9] in materials including stainless steels and a nickel-based superalloy, is particularly prominent in titanium alloy [10,11,12]. A clear inflection point typically appears at a certain strain amplitude, resulting in two different slopes in the high- and low-strain-amplitude regimes. This phenomenon implies that a simplistic extrapolation of high-strain-amplitude data to predict fatigue life under real service conditions would lead to a significant overestimation, thereby posing a severe safety risk [13]. Therefore, elucidating the underlying physical mechanism of this bilinear behavior has become a central scientific challenge that must be addressed for accurate LCF life prediction of this alloy.

In an effort to explain this phenomenon, researchers have provided crucial insights from the perspective of microstructural evolution. Experimental observations have revealed fundamental differences in the fatigue damage modes at high and low strain amplitudes: at low strain amplitudes, cracks typically initiate from a single, highly localized stress concentration, with narrower fatigue striations. In contrast, at high strain amplitudes, multiple crack initiation sites are formed, and the fatigue striations are wider [13]. This difference is directly related to the micro-scale deformation mechanisms; for instance, dislocation motion is characterized by planar slip at low strain amplitudes, transitioning to more diffuse dislocation tangles at higher strain amplitudes [5]. Cruzado et al. [6] further proposed that the core of this transition lies in the evolution of plastic deformation homogeneity: as strain amplitude increases, plastic deformation transitions from a highly localized state to a more uniformly distributed pattern. This “evolution of heterogeneity” hypothesis has also been corroborated in other materials exhibiting bilinear behavior, such as nickel-based superalloys [7,14] and aluminum alloys [8], indicating its potential as a general mechanism.

While these studies provide critical qualitative perspectives, they are largely based on experimental observations and post-mortem analysis. They lack a theoretical framework capable of prospectively predicting and quantitatively unveiling the underlying driving forces. Traditional macroscopic phenomenological models, with their inherent “black-box” nature, cannot capture the complex fatigue behavior governed by micro-physical processes such as grain orientation and slip activity [15]. Consequently, there is an urgent need to develop a “micromechanics-damage coupled” model that can simulate microstructural evolution and incorporate a physics-based damage criterion, thereby elucidating the root cause of the bilinear phenomenon at a mechanistic level.

To address this challenge, this paper proposes a numerical simulation framework based on the Crystal Plasticity Finite Element Method (CPFEM). CPFEM is an ideal tool for investigating micromechanical behavior, as it can resolve the stress–strain response and plastic slip evolution at the grain scale under cyclic loading [16,17,18]. Building upon this, we further integrate the physical damage process with the micro-scale deformation field via a User-Defined Damage Initiation (UDMGINI) subroutine [19,20,21], thereby establishing a “virtual testing” platform capable of directly predicting fatigue crack initiation life. Based on this framework, the primary objectives of this study are:

1. Model Development and Validation: To develop and validate a high-fidelity CPFEM model that can accurately reproduce the cyclic mechanical response and LCF life of Ti-6Al-4V, including its bilinear characteristic.
2. Quantitative Mechanistic Analysis: To systematically compare the evolution of micro-scale plastic strain fields under high and low strain amplitudes using this model, and to quantitatively characterize the evolution of deformation heterogeneity from a statistical perspective by introducing the coefficient of variation (CV) as a key metric.
3. Exploration of Physical Origins: To investigate the relationship between the evolution of deformation heterogeneity and the macroscopic energy response through a multi-scale energy dissipation analysis, aiming to elucidate the underlying driving mechanism of the bilinear phenomenon from a physical standpoint.

This study aims to provide new physical insights into the LCF damage mechanism of Ti-6Al-4V and to lay a solid theoretical foundation for the development of mechanism-based life prediction models.

## 2. Theoretical Framework and Computational Methods

To investigate the bilinear Low Cycle Fatigue (LCF) behavior of Ti-6Al-4V alloy at the micro-mechanistic level, a crystal plasticity finite element (CPFE) model coupled with a physics-based damage criterion was developed. This framework consists of three core components: a crystal plasticity constitutive model to describe plastic deformation at the grain scale, a Representative Volume Element (RVE) to represent the material’s microstructural features, and a damage criterion to predict fatigue crack initiation.

### 2.1. Crystal Plasticity Constitutive Model

This study employs a rate-dependent crystal plasticity constitutive model that incorporates both isotropic softening and kinematic hardening to accurately capture the complex mechanical response of Ti-6Al-4V alloy under cyclic loading. The model is based on the classical theory of dislocation slip [22,23]. Its kinematic framework is founded on the multiplicative decomposition of the deformation gradient **F** into an elastic part, $F^{e}$, and a plastic part, $F^{p}$:

(1) $${F\;=\;F}^{e}F^{p}$$

Plastic deformation is assumed to be driven exclusively by shear slip on discrete crystallographic slip systems. The plastic velocity gradient, $L^{P}$, can thus be expressed as:

(2) $$L^{P}=\overset{N}{\underset{\alpha =1}{\sum }}{\dot{\gamma }}^{\left(\alpha \right)}s^{*\left(\alpha \right)}⊗n^{*\left(\alpha \right)}$$

where *N* is the total number of slip systems, ${\dot{\gamma }}^{\left(\alpha \right)}$ is the shear slip rate on the α-th slip system, and $s^{*\left(\alpha \right)}$ and $n^{*\left(\alpha \right)}$ are the slip direction and slip plane normal vectors in the reference configuration, respectively.

To accurately capture the Bauschinger effect, a kinematic hardening term (back stress, $X^{\alpha }$) is introduced into the flow rule originally proposed by Hutchinson [24]. The shear slip rate ${\dot{\gamma }}^{\alpha }$ on the α-th slip system is therefore determined by a modified power-law flow rule:

(3) $${\dot{\gamma }}^{\alpha }={\dot{\gamma }}_{0}\mathrm{sgn}\left(\tau^{\alpha }{\;-\;X}^{\alpha }\right){\left|\frac{\tau^{\alpha }{\;-\;X}^{\alpha }}{\tau_{c}^{\alpha }}\right|}^{m}$$

where ${\dot{\gamma }}_{0}$ is the reference shear strain rate, *m* is the strain rate sensitivity exponent, and $\tau^{\alpha }$ is the resolved shear stress (RSS) on the α-th slip system. The effective stress, $\tau^{\alpha }\;{-\;X}^{\alpha }$, drives the plastic flow. $\tau_{c}^{\alpha }$ is the critical resolved shear stress (CRSS) of the α-th slip system, whose evolution directly governs the macroscopic mechanical response of the material. The evolution of $\tau_{c}^{\alpha }$ and $X^{\alpha }$, which represent the isotropic hardening/softening and kinematic hardening behaviors, respectively, is central to the constitutive model.

#### 2.1.1. Isotropic Softening

Ti-6Al-4V is a typical cyclic softening material. Its softening primarily originates from the dissolution or refinement of metastable Ti_3_Al strengthening precipitates, which are repeatedly sheared by dislocations during cyclic plastic deformation. This mechanism, often referred to as “shear-softening” [25,26,27], leads to a reduction in the material’s overall yield strength. In the model, this is captured by the evolution of the isotropic slip resistance $\tau_{c}^{\alpha }$ (i.e., the CRSS). To precisely model this key physical process, this study adopts the softening model proposed by Marano et al. [28]. This model assumes that the slip resistance first undergoes a rapid exponential decay, followed by a slow linear softening stage. The evolution law is given by:

(4) $$\tau_{c}^{\alpha }=\tau_{c_{i}}^{\alpha }-\Delta \tau^{\alpha }\left(1-\exp\left(-\frac{\gamma_{\mathrm{cum}}^{\alpha }}{\gamma_{0}^{\alpha }}\right)\right)+H\cdot \gamma_{\mathrm{cum}}^{\alpha }$$

where $\tau_{c_{i}}^{\alpha }$ is the initial slip resistance; $\Delta \tau^{\alpha }$ is the saturation value of cyclic softening, representing the maximum achievable softening in the exponential stage; $\gamma_{\mathrm{cum}}^{\alpha }$ is the accumulated plastic slip on the α-th slip system; $\gamma_{0}^{\alpha }$ is a reference slip controlling the rate of exponential softening; and *H* is a constant describing the slope of the subsequent linear softening stage. For a purely softening material, *H* is typically a small negative value or zero.

#### 2.1.2. Kinematic Hardening

Kinematic hardening describes the translation of the yield surface center in stress space and is primarily used to capture the early reverse yielding upon load reversal (the Bauschinger effect) and the transient hardening behavior in the initial cycles [29,30]. This study employs the classic Armstrong–Frederick (A–F) non-linear kinematic hardening model [31] to describe the evolution of the back stress, $X^{a}$:

(5) $${\dot{X}}^{a}=h{\dot{\gamma }}^{a}-h_{D}X^{a}\left|{\dot{\gamma }}^{a}\right|$$

where ${\dot{X}}^{a}$ is the evolution rate of the back stress, h is the initial kinematic hardening modulus that determines the initial slope of the back stress evolution, and $h_{D}$ is a dynamic recovery coefficient that allows the back stress to saturate during cyclic loading. The saturation value of the back stress is determined by the ratio h /$\;h_{D}$.

By coupling the aforementioned isotropic softening (Equation (4)) and kinematic hardening (Equation (5)) models into the flow rule (Equation (3)), a comprehensive crystal plasticity constitutive model capable of describing the cyclic softening, Bauschinger effect, and strain rate sensitivity of Ti-6Al-4V was constructed. The model was implemented in ABAQUS/Standard via a user material subroutine (UMAT).

### 2.2. Construction and Validation of the Representative Volume Element (RVE)

**Figure 1:**
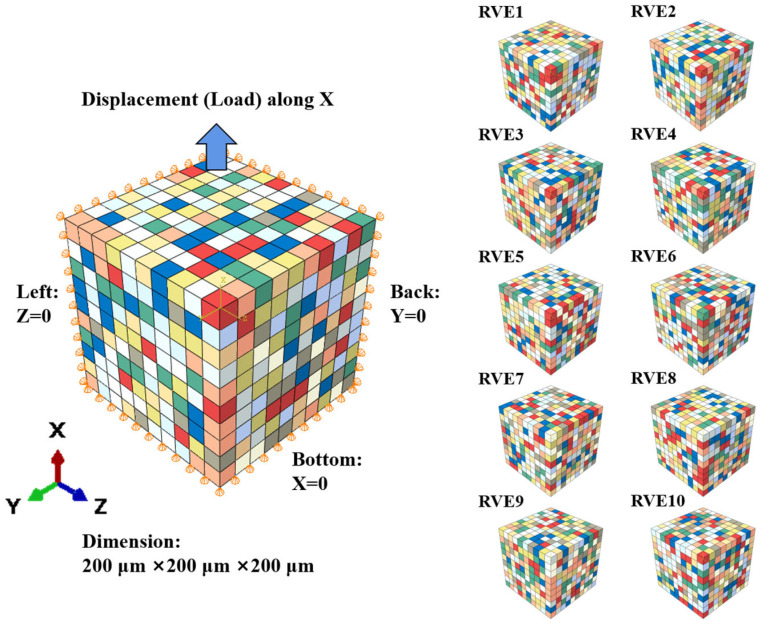
A schematic diagram of the RVE model composed of 1000 units, with RVE1-10 representing ten instances of the RVE with different random crystallographic orientation distributions to ensure statistical representation. Colors represent crystallographic orientation variations.

To simulate the mechanical behavior of polycrystalline Ti-6Al-4V while balancing the significant computational demands of LCF simulations, a simplified three-dimensional RVE was constructed and validated. The as-received Ti-6Al-4V alloy is a typical α+β dual-phase alloy. However, given its low β-phase volume fraction (<9%) and supported by multiple studies [32,33,34] confirming its negligible influence on the overall macroscopic mechanical response in this context, a single-phase RVE composed solely of equiaxed α-grains was constructed. This approach allows us to focus on the damage evolution in the dominant phase while ensuring the computational feasibility of LCF simulations. The RVE model, shown in Figure 1, consists of an aggregate of 1000 cubic elements (10 × 10 × 10) using eight-node linear brick elements (C3D8). 

In this model, a computationally efficient “one-element-per-grain” strategy was adopted. Each C3D8 element was assigned an independent crystallographic orientation, effectively representing a single grain. The edge length of each element was set to 20 μm, which corresponds to the average grain size of the equiaxed α-phase in this batch of Ti-6Al-4V alloy, as observed experimentally [26]. The primary advantage of this strategy is its high computational efficiency, which makes it possible to perform full-process simulations of LCF behavior over a high number of cycles (>5000 cycles).

To validate the statistical robustness of this simplified approach, 10 independent RVE models were generated. Each model contains 1000 elements (grains), but their crystallographic orientations were generated using different random seeds to represent 10 distinct microtextured samplings, as shown in Figure 1. Through subsequent parameter calibration and comparative analysis (see Section 2.3), we demonstrated that all 10 RVE sets yield highly consistent macroscopic mechanical responses under identical loading conditions. This indicates that although the RVE model is simplified, it is sufficient to stably capture the material’s average macroscopic behavior, which is governed by inter-granular interactions, and its predictions are independent of specific random orientation distributions.

To perform virtual LCF tests on the constructed RVE, the following boundary and loading conditions were applied. All simulations were conducted under total strain control with a fully reversed strain ratio (R = −1), and the loading direction was along the X-axis. To eliminate rigid body motion [33], normal displacement constraints were applied to the left, bottom, and back faces of the RVE, as shown in Figure 1.

### 2.3. Model Parameter Calibration and Validation

The predictive accuracy of a CP model is highly dependent on the precise calibration of its material parameters. The macroscopic experimental data required for calibration and validation in this study were all sourced from the authors’ previous systematic experimental research on the same batch of Ti-6Al-4V alloy [26]. In that work, low cycle fatigue experiments were carried out with a total strain-controlled mode, with a ratio of −1, a constant total strain rate of 4 × 10^−3^ s^−1^ and a triangular waveform performed on a computer-controlled 250 kN MTS810 closed-loop servo-hydraulic test machine (MTS Systems Corporation, Eden Prairie, MN, USA) at room temperature. Strain control was achieved by an extensometer (12 mm gage length) with its arm tips located on the gage length. The strain amplitude chosen for the present tests ranges from 0.7% to 2.0%. The tests were continued until fracture.

#### 2.3.1. Establishment of Elastic Constants

For the HCP structure of α-Ti, the independent single-crystal elastic constants are C_11_, C_12_, C_13_, C_23_, C_33_, C_44_ and C_66_. We used experimental values reported in the literature [34] as an initial reference and then fine-tuned these constants by simulating the macroscopic elastic response. This was done to ensure that the macroscopic Young’s modulus (E) predicted by the 1000-grain RVE accurately matched the elastic data measured during the initial stage of tensile tests on this material batch [26]. The finally determined elastic constants are listed in Table 1.

#### 2.3.2. Calibration of Plastic Parameters and Model Validation

A hierarchical, physics-informed approach was adopted for the calibration of plastic parameters to accurately capture the complex slip behavior of the HCP crystal.

First, to establish the initial relative strengths of the various slip systems (Basal, Prismatic, Pyramidal), we referenced nano-indentation data from [34], which provided activation stresses for each slip system. This allowed us to set the physical ratios between their initial CRSS values.

Subsequently, with these relative strengths fixed, the entire parameter set—including a base slip resistance value along with the isotropic softening ($\Delta \tau^{\alpha }$, $\gamma_{0}^{\alpha }$, *H*, ${\dot{\gamma }}_{0}$, *m*) and kinematic hardening ($h,\;\;h_{D}$) parameters—was systematically optimized by iteratively fitting the model’s response to both monotonic tensile and 1.8% strain amplitude cyclic experimental data [26].

A core feature of this calibration process is its dual-validation mechanism, which simultaneously verifies the accuracy of the parameters and the statistical robustness of the simplified RVE model. To this end, the same set of optimized plastic parameters was used to run validation simulations on all 10 independent RVEs with different random orientation distributions, as constructed in Section 2.2.

**Figure 2:**
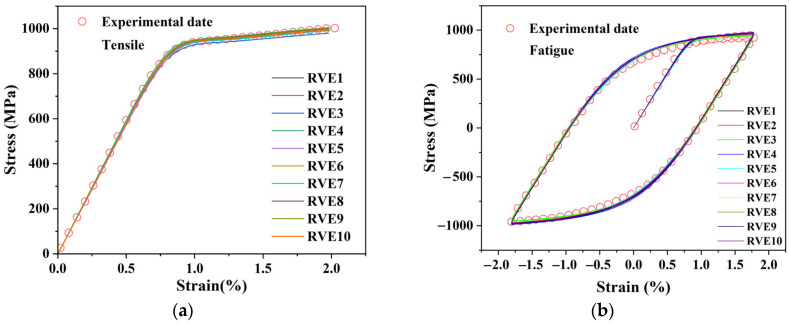
(**a**) Comparison of monotonic tensile curves; (**b**) Comparison of stabilized hysteresis loops at a strain amplitude of 1.8%. Experimental data are taken from [26], while the simulation results correspond to the response curves of 10 RVE with different random orientations.

Figure 2a,b present the key validation results. In the Figures, the simulated response curves (solid lines) for all 10 RVEs show excellent agreement with the experimental data (hollow circles) for both monotonic tension and 1.8% strain amplitude cycling. This result holds a dual significance: first, it confirms the accuracy of the calibrated plastic parameters; second, and more importantly, it demonstrates that the simplified “one-element-per-grain” RVE model used in this study is robust, as its macroscopic predictions are not sensitive to the random sampling of micro-orientations. The finally optimized plastic parameters are listed in Table 2.

**Figure 3:**
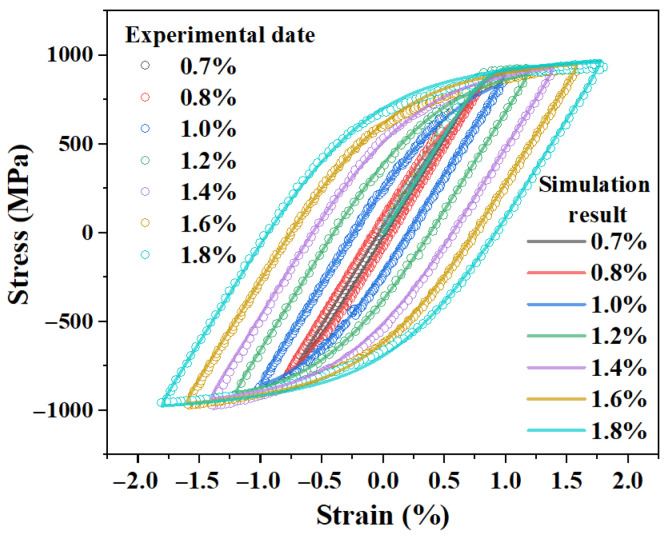
Comparison between simulated and experimental stabilized cyclic responses at different strain amplitudes.

As a final confirmation of the parameter set’s applicability, one of the RVE models was used to simulate the cyclic response at several different strain amplitudes (from 0.7% to 1.8%). As shown in Figure 3, the predicted initial hysteresis loops remained in good agreement with the corresponding experimental data [26]. This demonstrates that the calibrated parameter set possesses excellent extrapolation capabilities, which lays a solid foundation for the subsequent fatigue life analysis.

### 2.4. Framework for Fatigue Crack Initiation and Propagation

To directly simulate and predict fatigue crack initiation within the Crystal Plasticity Finite Element (CPFEM) framework, this study adopts an approach that integrates micromechanical physics with damage mechanics. This is achieved by coupling a User-defined Material subroutine (UMAT) with a User-defined Damage Initiation subroutine (UDMGINI) in Abaqus, leveraging the eXtended Finite Element Method (XFEM) to handle the crack discontinuity. This approach requires two distinct components: a criterion to determine when a crack initiates, and a law to govern how the damage evolves and propagates afterward.

#### 2.4.1. Criterion for Crack Initiation

The physical origin of LCF damage in Ti-6Al-4V lies in the irreversible accumulation of dislocation slip within grains, driven by cyclic plastic deformation—a process often manifested by the formation of Persistent Slip Bands (PSBs) [35,36]. Therefore, an effective Fatigue Indicator Parameter (FIP) must be capable of directly quantifying this core mechanism.

To select an FIP that is both physically meaningful and numerically robust, we referenced the systematic comparative study by Abdolvand et al. [37]. This study evaluated the performance of four different micro-damage models (based on principal stress, principal plastic strain, maximum slip, and energy stored in dislocations) in predicting crack initiation and propagation in HCP crystals. Their findings conclusively demonstrated that among the compared models, the criterion based on maximum cumulative slip (MAXSLP) not only predicted the initiation site of the primary crack most accurately but also most effectively reproduced its correct propagation direction along the slip plane. Based on this strong evidence, this study selects the maximum accumulated plastic slip on a slip system as the FIP to drive fatigue crack initiation.

Within the Abaqus damage mechanics framework, the UDMGINI subroutine uses a dimensionless scalar damage indicator variable, *f*, to determine material failure. Based on the chosen FIP, this variable is defined as [37]:

(6) $$f=\frac{\mathrm{Max}\left(\Gamma^{\alpha }\right)}{\Gamma_{crit}}$$

where *Γ*^α^ is the absolute value of the cumulative slip on the α-th slip system, and $\Gamma_{crit}$ is the critical cumulative slip at which a crack nucleates. Crack initiation occurs when *f* reaches 1.0. The normal to the crack plane is assumed to be parallel to the normal of the most active slip system (i.e., the predominant slip system) that causes crack nucleation.

#### 2.4.2. Law for Damage Evolution and Propagation

Once a crack has initiated in an element (i.e., when *f* = 1.0), its subsequent growth and the associated degradation of the element’s load-carrying capacity are governed by a damage evolution law.

In this study, we employ an energy-based damage evolution model, which is physically consistent and helps to mitigate mesh sensitivity. This approach is founded on the concept of fracture energy (*G*_c_), which represents the energy required to create a new unit area of crack surface. The material’s response after damage initiation is described by a traction-separation law, where the degradation is controlled by a scalar damage variable, *D*, which evolves from 0 (undamaged) to 1 (fully failed). The stress components (*t*) in the damaged element are progressively reduced according to the relation [37]:

(7) $$t=\left(1-D\right)T$$

where *T* represents the stress predicted by the elastic-plastic response in the absence of damage. The evolution of *D* is driven by the energy dissipated during the failure process, ensuring that the total energy dissipated to fail an element is equal to the prescribed fracture energy, *G*_c_. This law dictates how the element softens and fails, allowing the macroscopic crack to advance through the microstructure.

#### 2.4.3. Calibration of Damage Model Parameters

The coupled damage model contains two critical parameters that govern the material’s fatigue response: the critical cumulative slip (*Γ*_crit_) for crack initiation, and the fracture energy (*G*_c_) for damage evolution and crack propagation. The total fatigue life, *N*_f_, is the sum of the initiation life (*N*_ini_), which is primarily controlled by *Γ*_crit_, and the propagation life (*N*_p_), which is primarily controlled by *G*_c_. Consequently, these two parameters are interdependent and must be calibrated simultaneously to accurately capture the total fatigue life.

To perform the calibration, the experimental fatigue life at a strain amplitude of *ε*_ta_ = 1.2% was chosen as the benchmark target. This choice was made to ensure the physical representativeness and general applicability of the calibrated parameter. We acknowledge that experimental fatigue data inherently contains scatter, which can arise from minute variations in specimen surface finish, local microstructure, and testing conditions. We avoided excessively high strain amplitudes (e.g., 1.8%), where damage mechanisms might be convoluted with quasi-static fracture, as well as excessively low strain amplitudes, where experimental life is highly sensitive to minute surface defects, leading to greater data scatter. In contrast, the intermediate strain amplitude of 1.2% falls within a stable LCF regime where damage is dominated by the internal accumulation of cyclic plasticity within grains, providing a robust and reliable benchmark for parameter calibration. The calibration was performed through an iterative process of simultaneously adjusting the (*Γ*_crit_, *G*_c_) pair until the simulation results satisfied the criteria: the predicted total fatigue life (*N*_f_) quantitatively matched the experimental value.

In addition to calibrating fatigue life, it is crucial to define the physical crack orientation. Based on the underlying slip-driven failure mechanism, the model dictates that once the initiation criterion (*f* = 1.0) is met, the normal to the newly formed crack plane is defined to be parallel to the normal of the specific slip system (α) that reached the critical cumulative slip. This ensures the crack propagates along a physically meaningful crystallographic path.

Through this physically-grounded, coupled calibration process, the optimal parameter set was determined to be:

Critical Cumulative Slip, *Γ*_crit_ = 0.06.

Fracture Energy, *G*_c_ = 0.589 J/m^2^.

Crucially, this single set of calibrated parameters was then held constant and used for all subsequent predictive simulations across the entire range of strain amplitudes. This rigorous approach allows for a true test of the model’s ability to predict the bilinear Coffin-Manson behavior without any further parameter tuning.

## 3. Results and Discussion

### 3.1. Model Validation: From Microscopic Damage Modes to Macroscopic Life Prediction

To investigate the origin of the bilinear characteristic in the Coffin-Manson (C-M) curve of the Ti-6Al-4V alloy, it is essential to first validate that the coupled CPFEM-XFEM model can accurately capture the key physical phenomena of fatigue damage, from microscopic crack dynamics to macroscopic life prediction.

**Figure 4:**
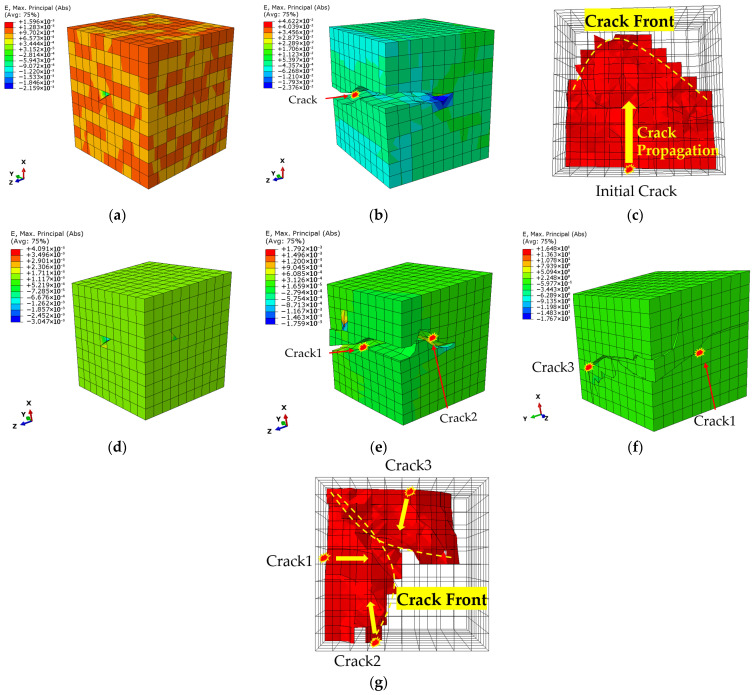
Detailed comparison of simulated fatigue damage evolution. Low Strain (0.7%): (**a**) Single crack initiation, (**b**) Final failure state, (**c**) Top-down view of the single, semi-elliptical crack face at failure. High Strain (1.8%): (**d**) Multiple crack initiation, (**e**) Coalescence of Cracks 1 & 2, (**f**) View showing non-coalesced Cracks 1 & 3, (**g**) Top-down view of the two final crack faces at failure.

Figure 4 provides a detailed, comparative visualization of the entire simulated fatigue failure process at low (0.7%) and high (1.8%) strain amplitudes. The results reveal a fundamental difference in the damage mechanism.

At the low strain amplitude of 0.7%, the process is characterized by singular, contained damage (Figure 4a–c). A single microcrack initiates on the RVE surface (Figure 4a). This crack then propagates steadily until final failure, which is defined by the sudden drop in cyclic stress (Figure 4b). A top-down perspective view, isolating the crack face at the moment of failure (Figure 4c), shows that the crack front maintains a regular, semi-elliptical arc. At the point of failure, this single crack has grown to occupy approximately three-quarters of the model’s cross-sectional area. This behavior is indicative of a classic, propagation-controlled failure originating from a single critical stress concentration.

In stark contrast, the high strain amplitude of 1.8% triggers a distributed, multi-site damage process (Figure 4d–g). The simulation predicts the initiation of three independent microcracks on the surface (Figure 4d, showing two of the initiation sites). The subsequent propagation is complex and interactive. Figure 4e shows that during propagation, two of the cracks (Crack 1 and Crack 2) merge to form a single, larger crack front. However, the third crack propagates independently and does not coalesce with the others, as shown from a different viewing angle in Figure 4f. The final failure state, therefore, consists of two separate, large cracks. This is confirmed by the top-down perspective view in Figure 4g, which isolates the final fracture surfaces. Critically, the combined area of these cracks at failure constitutes approximately two-thirds of the total cross-section—smaller damage volume than in the low-strain case.

**Figure 5:**
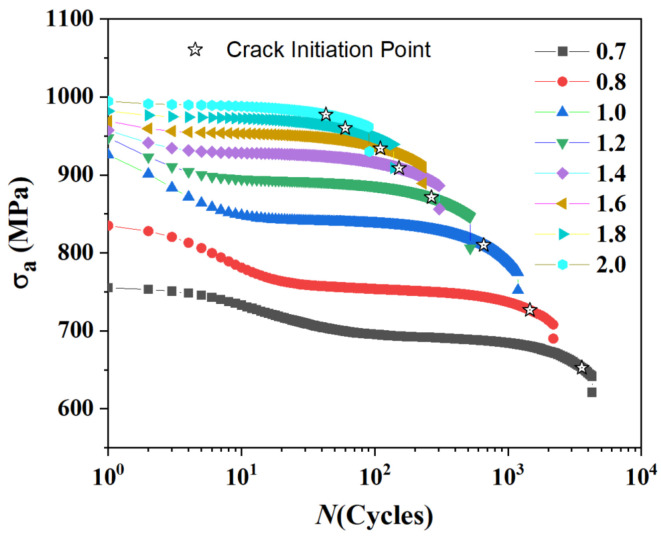
A Cyclic stress response curve of Ti-6Al-4V material. The stars (☆) indicate the simulated crack initiation life (*N*_ini_).

**Figure 6:**
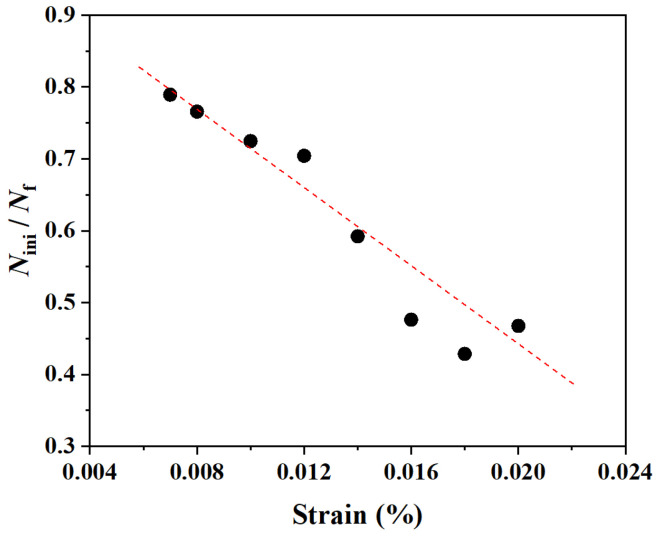
Ratio of crack initiation life (*N*_ini_) to total failure life (*N*_f_) as a function of total strain amplitude.

This stark difference in damage accumulation dynamics directly explains the observed trend in fatigue life partitioning. In Figure 5, the cyclic stress response curves are shown with the crack initiation life (*N*_ini_) explicitly marked. Plotting the ratio of initiation life to total life (*N*_ini_/*N*_f_) in Figure 6 reveals that the *N*_ini_/*N*_f_ ratio decreases from ~0.8 at low strain to ~0.45 at high strain. At low strain, the long propagation phase of a single crack dominates the post-initiation life. At high strain, the rapid growth and coalescence of multiple cracks lead to a much shorter propagation phase relative to the total life.

This difference in damage mode is also reflected in the temporal evolution of the fatigue process. Figure 5 shows the cyclic stress response curves, where we have explicitly marked the crack initiation life (*N*_ini_)—the number of cycles for the first element to fail—for each strain amplitude. The total failure life (*N*_f_) is defined by the final sharp stress drop. To quantify the relationship between these two stages, the ratio of initiation life to total life (*N*_i_/*N*_f_) is plotted in Figure 6.

Two clear trends emerge from this analysis. First, the *N*_ini_/*N*_f_ ratio systematically decreases as the strain amplitude increases, falling from approximately 0.8 at 0.7% strain to about 0.45 at 1.8% strain. This quantitatively confirms that low-cycle fatigue at low strain amplitudes is initiation-dominated, while the propagation stage becomes progressively more significant at higher strain amplitudes. Second, the predicted ratio range of 0.45–0.8 is in good agreement with experimental observations reported for similar alloys [38].

**Figure 7:**
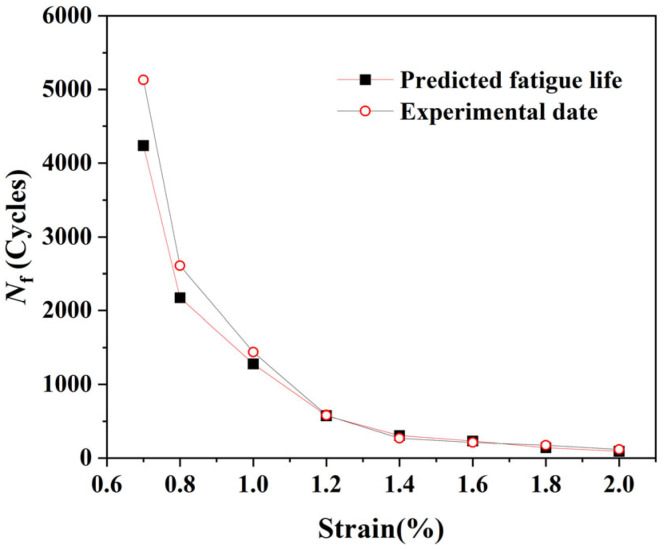
Comparison of predicted low-cycle fatigue life with experimental data for Ti-6Al-4V alloy.

Finally, the model’s ultimate predictive capability is assessed in Figure 7, which compares the predicted total fatigue lives (*N*_f_) with experimental data [25]. The results show excellent agreement across the entire strain amplitude range, with all prediction points falling within the factor-of-two scatter band.

In summary, the established CPFEM-XFEM framework is validated on three critical levels: it reproduces realistic, strain-dependent microscopic failure modes (Figure 4); it correctly captures the relative importance of the initiation and propagation stages (Figure 6); and it achieves high-precision quantitative prediction of the total fatigue life (Figure 7). This lays a solid and reliable foundation for the subsequent analysis.

### 3.2. Bilinearity of the C-M Curve

**Figure 8:**
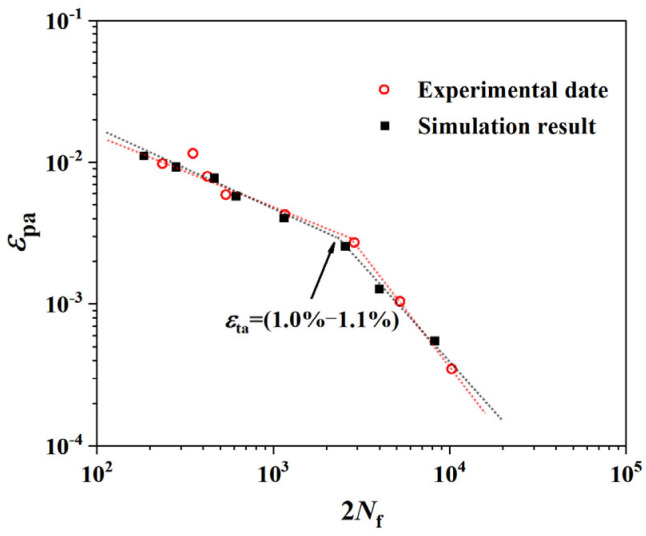
Coffin-Manson curve of Ti-6Al-4V alloy.

Figure 8 compares the Coffin-Manson (C-M) curve predicted by the model with experimental data [26], showing excellent agreement over the entire life range. Crucially, the coupled model developed herein successfully reproduces the bilinear characteristic of the C-M relationship observed in experiments without introducing any ad-hoc assumptions.

In the log–log plot, the life curve exhibits a distinct transition point, or “knee,” in the region of *ε*_ta_ = (1.0–1.1%). To the left of this point, in the high strain amplitude regime (*ε*_ta_ ≥ 1.2%), the slope of the curve is relatively shallow. To the right, in the low strain amplitude regime (*ε*_ta_ < 1.2%), the slope becomes steeper.

This result strongly demonstrates that our crystal plasticity model, coupled with a physics-based damage criterion, can intrinsically capture the key factors that cause the transition in the macroscopic fatigue behavior of Ti-6Al-4V. The bilinear phenomenon is not an extrinsic representation obtained through parameter fitting but rather a natural consequence of the interplay between microscopic plastic deformation evolution and damage accumulation. This provides a firm basis for further investigation into its physical origins.

### 3.3. Qualitative Analysis of Microscopic Deformation Inhomogeneity

**Figure 9:**
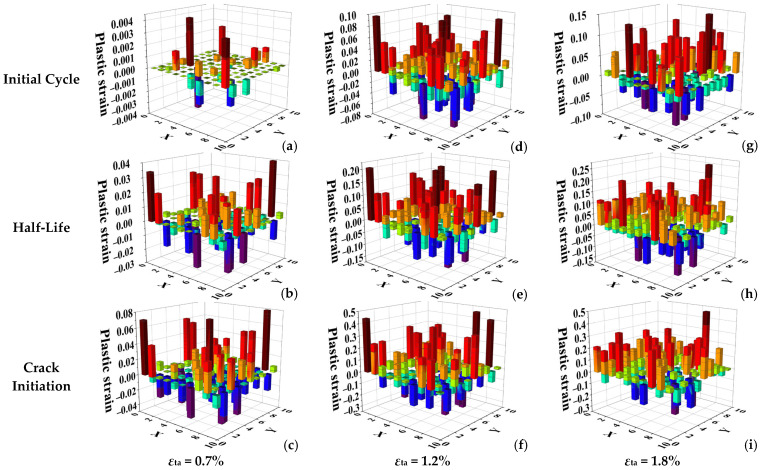
Histograms of the plastic strain distribution on the x-y plane at the time of initial cycle, half-life and crack initiation under different strain amplitudes: (**a**–**c**) 0.7%; (**d**–**f**) 1.2%; (**g**–**i**) 1.8%.

To reveal the micro-mechanisms behind the bilinear low-cycle fatigue behavior of Ti-6Al-4V, this section qualitatively analyzes the inhomogeneity of grain-scale plastic deformation and its evolution at different strain amplitudes. Figure 9 presents histograms of the plastic strain distribution on an x–y cross-section of the RVE model at three strain amplitudes (0.7%, 1.2%, and 1.8%) and at different life stages (initial cycle, half-life, and crack initiation). These histograms allow for a qualitative assessment of the degree of plastic deformation inhomogeneity. It is evident that in the low strain amplitude regime (0.7%), the plastic strain distribution is highly heterogeneous, with concentrated plastic deformation occurring in a few grains. In contrast, in the high strain amplitude regime (1.8%), the plastic strain distribution is more uniform, with a large number of grains participating in the plastic deformation.

Comparing the different life stages at low strain amplitude (Figure 9a–c), the plastic deformation exhibits a high degree of localization. The vast majority of plastic strain is concentrated within a few specific “soft-oriented” grains, while the surrounding “hard-oriented” grains undergo almost no plastic deformation. This indicates that damage accumulation is strictly confined to these “weak links.” In contrast, at high strain amplitude (Figure 9g–i), the plastic deformation displays significant homogenization. A large number of grains participate in the plastic deformation. Although strain levels still vary between grains, extreme strain concentration zones no longer exist, and the overall deformation is more evenly distributed. This transition from “localized concentration” to “widespread participation” suggests a fundamental change in the mode and efficiency of damage accumulation.

**Figure 10:**
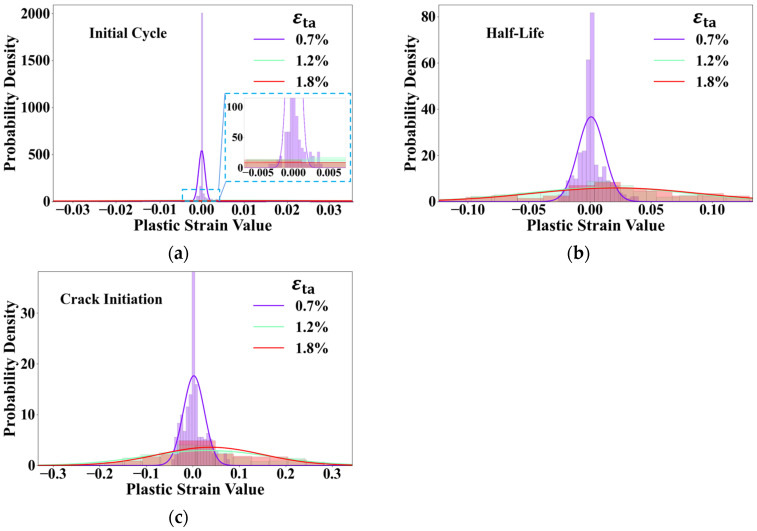
Normal distribution statistics graphs of the x-y plane of initial cycle, half-life, and crack initiation under different strain amplitudes: (**a**) initial cycle; (**b**) half-life; (**c**) crack initiation.

To further quantify the qualitative observations from Figure 9 from a statistical perspective, normal distribution fitting was performed on the plastic strain values for each cross-section, with the results shown in Figure 10. In statistics, the shape of a normal distribution curve directly reflects the dispersion of the dataset: a taller, narrower curve indicates that the data are concentrated around the mean, whereas a shorter, broader curve signifies a wider data distribution. In this study, at the low strain amplitude (0.7%), the distribution curves are tall and narrow at all life stages. This precisely quantifies the observation in Figure 9a–c: the plastic strain in the vast majority of grains is concentrated near zero (i.e., no plastic deformation), while a very small number of grains accommodate extremely high plastic strains, leading to a highly concentrated data distribution and thus, highly heterogeneous plastic deformation. In contrast, at high strain amplitudes (1.2% and 1.8%), the distribution curves are significantly shorter and broader. This indicates that plastic strain is more widely distributed among a larger number of grains. The increased participation of grains and the wider range of strain values statistically confirm the trend of plastic strain homogenization, meaning the deformation becomes more uniform.

This transition from “localized concentration” to “widespread participation” is the fundamental reason for the change in damage mode observed in Figure 4. Extreme localization at low strain amplitudes provides only one viable site for crack initiation, while homogenized deformation at high strain amplitudes creates multiple potential “weak links” across the material.

### 3.4. Quantification of Deformation Inhomogeneity

To quantitatively characterize the degree of plastic deformation inhomogeneity and reveal its intrinsic link to the bilinear behavior, this study employs the Coefficient of Variation (*CV*) [39,40]. The *CV* is the ratio of the standard deviation (*S*) to the absolute mean ($\bar{\varepsilon_{p}}$) (see Equations (8)–(10)). As a dimensionless metric, it eliminates the influence of scale and mean value differences, thereby enabling an unbiased comparison of deformation dispersion across different strain amplitudes. A higher *CV* value signifies a more dispersed and thus more heterogeneous plastic deformation distribution [26].

(8) $$CV=S/\left|\bar{\varepsilon_{p}}\right|$$

where ($\bar{\varepsilon_{p}}$) is the mean of the plastic strain distribution:

(9) $$\bar{\varepsilon_{p}}=\frac{1}{n}\overset{n}{\underset{i=1}{\sum }}\varepsilon_{p_{i}}$$

In these equations, $\varepsilon_{p_{i}}$ represents the plastic strain of the i-th finite element, and *n* is the total number of grains and *S* is the standard deviation, expressed as:

(10) $$S=\sqrt{\frac{1}{n-1}\overset{n}{\underset{i=1}{\sum }}{\left(\varepsilon_{p_{i}}-\bar{\varepsilon_{p}}\right)}^{2}}$$

**Figure 11:**
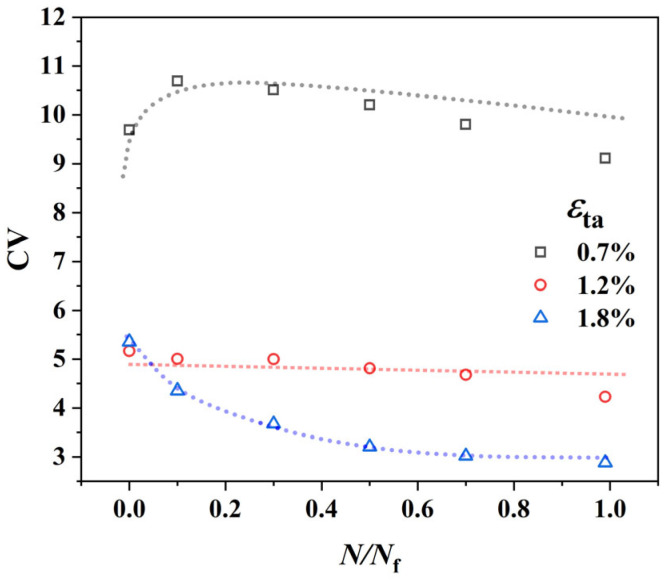
Evolution of the coefficient of variation (*CV*) of low-cycle fatigue plastic density under different strain amplitudes.

Figure 11 illustrates the evolution of the plastic strain *CV* within the RVE as a function of life fraction for three representative strain amplitudes. These curves clearly reveal that the evolutionary paths of deformation inhomogeneity are fundamentally different depending on the strain amplitude:

In the high strain amplitude regime (*ε*_ta_ = 1.8%): The *CV* value exhibits an exponential decay, rapidly decreasing from an initial value of 5.5 to approximately 3.0. This indicates that, driven by the high strain, the system quickly self-accommodates by activating a large number of slip systems, leading to the homogenization of plastic deformation.

In the low strain amplitude regime (*ε*_ta_ = 0.7%): The evolution pattern is starkly different. In the early cycles, the *CV* value rapidly climbs from an initial 9.7 to a very high level (10.8) before slowly decreasing. Nevertheless, it remains at a level significantly higher than that of the other strain amplitudes throughout the entire fatigue life. This “anomalous” initial increase reflects the extreme localization of deformation at low strain amplitudes: a few “soft” grains are “activated” in the early cycles, causing a sharp increase in the strain disparity between them and the vast majority of “dormant” grains, thus elevating the *CV* value.

At the transition point (*ε*_ta_ = 1.2%): As the “knee” of the C-M curve, its *CV* evolution also displays transitional behavior. The *CV* value remains at an intermediate level of around 5.0 and shows a slight decreasing trend with cycling.

This finding, which shows excellent agreement with the experimental measurements reported in [26], corroborates the physical fidelity of our model. It clearly indicates that the difference in mechanical response between high and low strain amplitudes is rooted in these two distinct modes of micro-deformation evolution. This evolution of heterogeneity is the direct cause of the transition from single-site to multi-site damage, which in turn leads to a deeper question: How do these different evolutionary paths affect the energy dissipation within the material and ultimately determine its fatigue life?

### 3.5. The Duality of Energy Dissipation: From Microscopic Conservation to Macroscopic Variability

To dissect the damage mechanism from a more fundamental energy perspective, this section investigates the evolution of plastic energy dissipation at both the local (grain) and global (RVE) scales. The primary local metric is the Cumulative Plastic Strain Energy Density (*W*_p_), which represents the total plastic work dissipated per unit volume within a single material point (i.e., a grain in this model). It is calculated by integrating the plastic power over time:

(11) $$W_{p}=\underset{\alpha }{\sum }\;\int_{0}^{t}\;\tau^{\alpha }{\dot{\gamma }}^{\alpha }dt$$

To capture the macroscopic energy response of the entire aggregate, the volume-averaged Cumulative Plastic Strain Energy Density across all grains in the RVE, denoted as $\bar{W_{p}}$, is also calculated:

(12) $$\bar{W_{p}}=\frac{1}{n}\overset{n}{\underset{i=1}{\sum }}W_{p}$$

**Figure 12:**
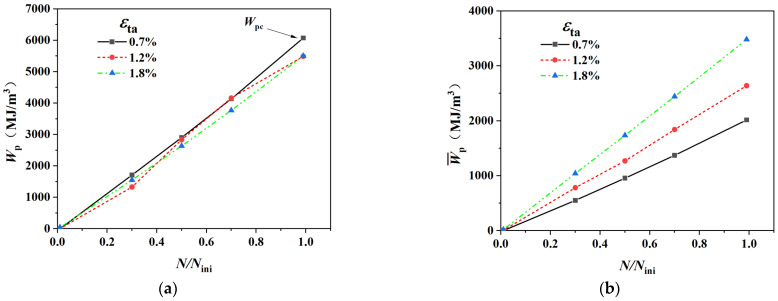
(**a**) Evolution of Plastic Strain Energy in Grains with Crack Initiation under Different Strain Amplitudes; (**b**) Evolution of the Average Plastic Strain Energy in All Grains under Different Strain Amplitudes.

This dual-scale energy analysis allows us to distinguish between the energy required to trigger failure in a single critical grain and the total energy dissipated by the entire material system, providing a direct link between microscopic inhomogeneity and macroscopic fatigue life. First, we examine the “origin” of damage—the first grain to fail. Figure 12a presents the evolution of *W*_p_ in the first-to-fail grain as a function of life fraction (*N/N*_ini_) for different strain amplitudes. A profound and unified microscopic law is revealed: for all strain amplitudes, the *W*_p_ exhibits a linear relationship with *N/N*_ini_. More importantly, not only do these three lines converge to the same critical energy value ($W_{\mathrm{pc}}$ as shown in Figure 12a) at the failure of the first-to-fail grain, but their slopes are also nearly identical. This finding implies that, at the micro-scale, not only is the total energy required to initiate a crack in a grain constant, but its rate of energy accumulation (relative to life fraction) is also independent of the macroscopic strain amplitude. It must be emphasized that this uniformity is not a preconceived condition of the model but a self-consistent outcome of the system’s evolution, strongly suggesting that fatigue crack initiation in Ti-6Al-4V follows an intrinsic and unified energy accumulation law.

However, when we shift our perspective from the microscopic “point” (a single grain) to the macroscopic “body” (the entire RVE), a completely different behavior emerges. As shown in Figure 12b, the average Cumulative Plastic Strain Energy Density for the entire RVE at the point of crack initiation (*N/N*_ini_ = 1), denoted as $\bar{W_{p}}$, is not uniform. Instead, it exhibits a pronounced strain-amplitude dependence, forming a clear hierarchy: $\bar{W_{p}}$ increases with increasing strain amplitude.

Thus, we observe a duality in energy dissipation: at the microscopic level, the energy accumulation law is uniform and conserved (Figure 12a); at the macroscopic level, the total energy dissipation is variable and dependent on loading conditions (Figure 12b).

The explanation for this duality is rooted in the differences in deformation inhomogeneity (*CV*) revealed in Section 3.4. The variability of the macroscopic average energy, $\bar{W_{p}}$, is a direct manifestation of the varying efficiency of energy transfer from the macroscopic input work to the microscopic critical damage:

In the low strain amplitude regime (high *CV*): Plastic deformation is highly localized, concentrating energy dissipation within a few grains. Consequently, the system requires a lower overall energy input (corresponding to a low $\bar{W_{p}}$ in Figure 12b) for one of these grains to be the first to reach the critical energy $W_{\mathrm{pc}}$. In this mode, the efficiency of energy transfer to damage “hotspots” is high. Consequently, the system requires a lower overall energy input for this single site to initiate a crack and propagate (as seen in Figure 4a–c).

In the high strain amplitude regime (low CV): Plastic deformation becomes more homogenized, distributing energy dissipation across a large number of grains. For any single grain to reach $W_{\mathrm{pc}}$, a substantial amount of energy must also be absorbed and dissipated by many non-critical grains, and by multiple growing crack fronts (as seen in Figure 4d–g). This leads to a massive total energy consumption by the system (corresponding to a high $\bar{W_{p}}$ in Figure 12b). In this mode, the energy transfer efficiency is low.

Herein, we complete the final argument for the bilinear damage mechanism. The bilinear C-M behavior of Ti-6Al-4V does not stem from two independent microscopic damage mechanisms. Instead, it is the inevitable manifestation of a single, unified microscopic energy-based damage law (the conservation of $W_{\mathrm{pc}}$ as shown in Figure 12a) operating under two distinctly different macroscopic energy dissipation modes (the variable $\bar{W_{p}}$, as shown in Figure 12b). The physical origin is as follows: different strain amplitudes drive different evolutionary paths of plastic deformation inhomogeneity. This, in turn, governs the energy transfer efficiency from the macroscopic work input to the microscopic damage sites, which ultimately presents itself as the “bilinear” macroscopic feature on the fatigue life curve.

## 4. Conclusions

This study conducted an in-depth investigation into the bilinear low-cycle fatigue damage mechanism of the Ti-6Al-4V alloy, employing a crystal plasticity finite element method coupled with a constitutive model that accounts for cyclic softening and a comprehensive damage model within a CPFEM-XFEM framework. Based on a quantitative analysis of the microscopic deformation fields, damage evolution, and energy dissipation at various strain amplitudes, the main conclusions are as follows:

1. Successful Reproduction and Validation: The developed crystal plasticity model successfully reproduced key fatigue characteristics of the Ti-6Al-4V alloy, including its cyclic stress response, hysteresis loops, and the bilinear Coffin-Manson (C-M) curve. The predicted fatigue lives show excellent agreement with experimental data, thereby validating the accuracy and effectiveness of the proposed coupled analysis framework.
2. Revealing the Unified Origin of Bilinearity: From Deformation Inhomogeneity to Failure Mode Transition: The origin of the bilinear behavior is a transition in the microscopic failure mode, which is directly driven by the evolution of plastic deformation inhomogeneity. At low strain amplitudes, the deformation remains highly localized (quantified by a high Coefficient of Variation, CV), concentrating damage accumulation within a few “hot-spot” grains. This confinement of damage naturally leads to the initiation and stable propagation of a single, dominant crack. Conversely, at high strain amplitudes, the deformation rapidly homogenizes (indicated by a low CV), creating multiple potential weak links across the microstructure. This distributed damage landscape results in a failure mode characterized by multi-site crack initiation and subsequent coalescence. Therefore, the evolution of deformation inhomogeneity is the direct cause of the shift in failure mode, which together constitute the unified physical origin of the macroscopic bilinear characteristic.
3. Underlying Physical Consequence: From an energy dissipation perspective, a “duality of damage” was identified: while the microscopic energy dissipation threshold ($W_{\mathrm{pc}}$) required for crack initiation in a single “critical” grain is conserved, the macroscopic average energy dissipation ($\bar{W_{p}}$) required for overall material failure is variable. This discrepancy is attributed to the different energy transfer efficiencies governed by the degree of deformation inhomogeneity (*CV*), which ultimately gives rise to the bilinear characteristic of the C-M curve.
4. Limitations and Outlook: The proposed “microscopic energy conservation” hypothesis is an inference drawn from a specific material and model; its universality requires further validation. Furthermore, the single-phase α-Ti approximation, while justified by the low β-phase fraction in the alloy, is a simplification. The presence of the β-phase could introduce local stress/strain concentrations at α/β interfaces, potentially influencing crack initiation sites. Similarly, this study was conducted under idealized conditions, excluding environmental factors such as oxidation and temperature, which could alter slip system activity and surface energy, thereby affecting both deformation heterogeneity and damage evolution. Future work should be directed towards two main areas: (i) computationally, extending the proposed framework to a wider range of metallic materials to test the generality of this energy law; and (ii) experimentally, leveraging high-resolution in situ characterization techniques to directly measure energy dissipation in real three-dimensional grains, which would provide definitive experimental support for the theory.

## Figures and Tables

**Table 1:** Calibration results of crystal plasticity on Ti-6Al-4V alloy parameters.

	Elastic Constants/GPa						
Phase 1	C_11_	C_12_	C_13_	C_23_	C_33_	C_44_	C_66_
α	139	79	69	68	167	41	29

**Table 2:** Rate-dependent viscoplasticity model and strain-hardening parameters.

$\Delta \tau^{\alpha }$ MPa	$\gamma_{0}^{\alpha }$	H MPa	${\dot{\gamma }}_{0}$ s^−1^	m	h MPa	$h_{D}$	$\mathrm{Strain-Hardening}\;\mathrm{Parameters}\;\tau_{0}$/MPa			
							Basal α	Prismatic α	Pyramidal α	1st/2nd Pyramidal c + α
115	0.004	−10	0.02	15	48,000	400	380	330	460	600

## Data Availability

Data can be provided upon request from the corresponding author.
